# Abnormal Cyclic Nucleotide Signaling at the Outer Mitochondrial Membrane In Sympathetic Neurons During the Early Stages of Hypertension

**DOI:** 10.1161/HYPERTENSIONAHA.121.18882

**Published:** 2022-05-04

**Authors:** Dan Li, Kun Liu, Harvey Davis, Calum Robertson, Oliver C. Neely, Adib Tarafdar, Ni Li, Konstantinos Lefkimmiatis, Manuela Zaccolo, David J. Paterson

**Affiliations:** Burdon Sanderson Cardiac Science Centre and BHF Centre of Research Excellence, Department of Physiology, Anatomy and Genetics (D.L., K.L., H.D., C.R., O.C.N., A.T., N.L., M.Z., D.J.P.), University of Oxford, United Kingdom.; Chinese Academy of Medical Sciences Oxford Institute (COI), Nuffield Department of Medicine Research Building (N.L.), University of Oxford, United Kingdom.; Department of Neuroscience, Physiology and Pharmacology, University College London, United Kingdom (H.D.).; Department of Molecular Medicine, University of Pavia, Italy (K.L.).; Veneto Institute of Molecular Medicine, Padova, Italy (K.L.).

**Keywords:** cyclic nucleotide, cytosol, hypertension, mitochondrial membrane, neurons, phenotype

## Abstract

Disruption of cyclic nucleotide signaling in sympathetic postganglionic neurons contributes to impaired intracellular calcium handling (Ca^2+^) and the development of dysautonomia during the early stages of hypertension, although how this occurs is poorly understood. Emerging evidence supports the uncoupling of signalosomes in distinct cellular compartments involving cyclic nucleotide–sensitive PDEs (phosphodiesterases), which may underpin the autonomic phenotype in stellate neurons.

**Methods:**

Using a combination of single-cell RNA sequencing together with Forster resonance energy transfer–based sensors to monitor cyclic adenosine 3’,5’-monophosphate, PKA (protein kinase A)-dependent phosphorylation and cGMP (cyclic guanosine 3’,5’-monophosphate), we tested the hypothesis that dysregulation occurs in a sub-family of PDEs in the cytosol and outer mitochondrial membrane of neurons from the stellate ganglion.

**Results:**

PDE2A, 6D, 7A, 9A genes were highly expressed in young Wistar neurons and also conserved in neurons from spontaneously hypertensive rats (SHRs). In stellate neurons from prehypertensive SHRs, we found the levels of cyclic adenosine 3’,5’-monophosphate and cGMP at the outer mitochondrial membrane were decreased compared with normal neurons. The reduced cyclic adenosine 3’,5’-monophosphate response was due to the hydrolytic activity of overexpressed PDE2A2 located at the mitochondria. Normal cyclic adenosine 3’,5’-monophosphate levels were re-established by inhibition of PDE2A. There was also a greater PKA-dependent phosphorylation in the cytosol and at the outer mitochondrial membrane in spontaneously hypertensive rat neurons, where this response was regulated by protein phosphatases. The cGMP response was only restored by inhibition of PDE6.

**Conclusions:**

When taken together, these results suggest that site-specific inhibition of PDE2A and PDE6D at the outer mitochondrial membrane may provide a therapeutic target to ameliorate cardiac sympathetic impairment during the onset of hypertension.

**Novelty and Relevance**

**What Is New?**

Cyclic nucleotide signaling is attenuated at the outer mitochondrial membrane (OMM) microdomain in sympathetic neurons during the early stages of hypertension. This impairment can be rescued by inhibition of local phosphodiesterase activity.
PKA (protein kinase A)-dependent phosphorylation is enhanced at OMM and rescued by phosphatase inhibition in sympathetic neurons from early stages of hypertension.

**What Is Relevant?**

Modulation of local phosphodiesterase activity and phosphatase in sympathetic neurons control the functional microdomains of cyclic nucleotide signaling at the OMM, which is likely to contribute to sympathetic dysautonomia.
Site-specific inhibition of local phosphodiesterase may provide a therapeutic target to ameliorate cardiac sympathetic impairment during the onset of hypertension.

**Clinical/Pathophysiological Implications?**

Using a combination of single-cell RNA sequencing, real time quantitative polymerase chain reaction, immunocytochemistry, together with forster resonance energy transfer–based OMM targeted sensors to monitor local cyclic nucleotide in sympathetic neurons, we have demonstrated that impairment of cAMP (cyclic adenosine 3’,5’-monophosphate)/PKA and cGMP (cyclic guanosine 3’,5’-monophosphate) at the OMM is modulated by site-specific actions of phosphodiesterase and phosphatase. These observations may provide an opportunity for the development of new approaches of targeted therapy to reduce or ablate stellate ganglia neuron activity in hypertension.

Autonomic dysfunction is a well-established component of essential hypertension in both humans and spontaneously hypertensive rats (SHRs)^1,2^—with dysautonomia preceding the onset of high blood pressure itself.^3,4^ Reduced parasympathetic drive^5^ and sympathetic hyperactivity^6,7^ both feature, with neurohumoral markers of sympathetic activity being elevated in hypertensive individuals.^8,9^ Crucially, sympathetic hyperactivity may result in a number of additional pathological consequences such as cardiac hypertrophy,^10^ arrhythmia,^11^ vascular dysfunction,^12^ and inflammation.^13^ Recently, focus has shifted to a more dominant role being played by the sympathetic nervous system in the etiology of hypertension.^14,15^

Cross-culture experiments have established that diseased sympathetic neurons from prehypertensive rats are powerful drivers of myocyte function, with evidence showing that healthy sympathetic neurons are able to rescue adrenergic function in diseased cardiomyocytes.^16^ Emerging data suggest that the diseased neuronal phenotype is underpinned by disruption of cyclic nucleotides signaling.^14^ In particular, the impaired hydrolysis of both cAMP (cyclic adenosine 3’,5’-monophosphate) and cGMP (cyclic guanosine 3’,5’-monophosphate) is linked to enhanced intracellular calcium transients and abnormal transmission.^17,18^ Cyclic nucleotides are compartmentalized and are coupled to specific cellular functions. Their local regulation relies on their hydrolysis by PDEs (phosphodiesterases), which also reside in cellular microdomains.^19^

Cyclic AMP regulates mitochondrial function in a cell-type specific manner. In particular, PDE2A is elevated in human stellectomised tissue from patients with sympathetic dysautonomia.^20^ PDE2A is a dual-substrate enzyme, able to hydrolyze both cAMP and cGMP with approximately equal affinity and efficacy.^21^ Overexpressing PDE2A in healthy sympathetic neurons recapitulates the decreased levels of cGMP^17^ associated with abnormal sympathetic transmission that is observed in prehypertensive rats, although its site of action in the neuron has not been established. Given the link between PDE2A, oxidative stress, and abnormal Ca^2+^ buffering, attention has focused on the role of the mitochondria as a putative site for sympathetic impairment.^14^ cAMP signaling at the outer mitochondrial membrane (OMM) has been shown to have diverse effects on mitochondrial function,^22,23^ including enhancement of the mitochondrial membrane potential (Ψm),^24^ which is more depolarised in the young spontaneously hypertensive rat (SHR). As major regulators of local cAMP levels, some PDE isoforms (including PDE2A2) have been shown to target specific mitochondrial features including morphology,^20^ clearance^21^ membrane potential, respiration, and permeability transition^22^; especially in cardiac mitochondria.^20,23^ In living cells, cytosolic cAMP and PKA (protein kinase A) is freely permeable from the cytosol to the OMM; however, the IMM is impermeable to cAMP.^25^ Moreover, AKAP (A-kinase anchor protein) on the OMM, and its phosphorylation by cytosolic PKA, suggests mitochondria might act as metabolic sensors^26^ that are under the influence of PDEs.

Therefore, we measured cytosolic and OMM levels of cAMP and PKA to investigate whether cyclic nucleotide microdomains are impacted in SHR neurons. Specifically, we tested the hypothesis that the mitochondrial localized isoform PDE2A2 underpins reduced cAMP and cGMP responses during the early development of dysautonomia in hypertension. We profiled PDE abundance in stellate ganglia using single-cell RNAseq and found evidence for PDE2A, and its isoform PDE2A2 being localized to the OMM. We also observed significant levels of expression of neuronal PDEs 4, 6, and 9. Using forster resonance energy transfer (FRET) sensors for cAMP and cGMP, we found the level of cAMP and cGMP at the OMM microdomain was decreased in SHR neurons, which was reversed by inhibition of PDE2A and PDE6D, respectively.

## Methods

An expanded Materials and Methods section is available in the Supplemental Material for neuronal culture methodology, RNAseq, qRT-PCR, FRET, and immunofluorescence microscopy. The single-cell RNA sequencing dataset generated during this study is available at genome expression omnibus (GSE144027). Further data and details on materials and protocols related to this study are also available upon reasonable request from the corresponding authors.

### Animals

Four-week-old male prehypertensive SHRs that have a well-established cellular sympathetic phenotype^20,27^ and normotensive Wistar rats were obtained from Envigo, United Kingdom and housed on a 12-hour day-night cycle. Animal use complied with the University of Oxford Local Ethical Guidelines and was in accordance with the Guide for the Care and Use of Laboratory Animals published by the US National Institutes of Health (Publication No. 85-23, revised 2011) and the Animals (Scientific Procedures) Act 1986 (United Kingdom). Experiments were performed under British Home Office Project License (PPL 30/3131 [D.J.P.] and P707EB251 [D.J.P.]). Animals were euthanized via an overdose of pentobarbitone and confirmed via exsanguination according to Schedule 1 of the Animals (Scientific Procedures) Act 1986 (United Kingdom).

### Statistical Analysis

Grouped data are expressed as mean±SEM, and all data passed a Shapiro-Wilk normality test. Unpaired Student *t*-tests were used to analyze the statistical significance of differences between 2 groups (Wistar and SHR). Statistical significance was accepted at *P*<0.05. n values indicate the number of neurons analyzed, and in each case represent neurons from at least 4 rats.

## Results

### Expression of PDE Gene Families in Sympathetic Neurons

**Figure 1.**
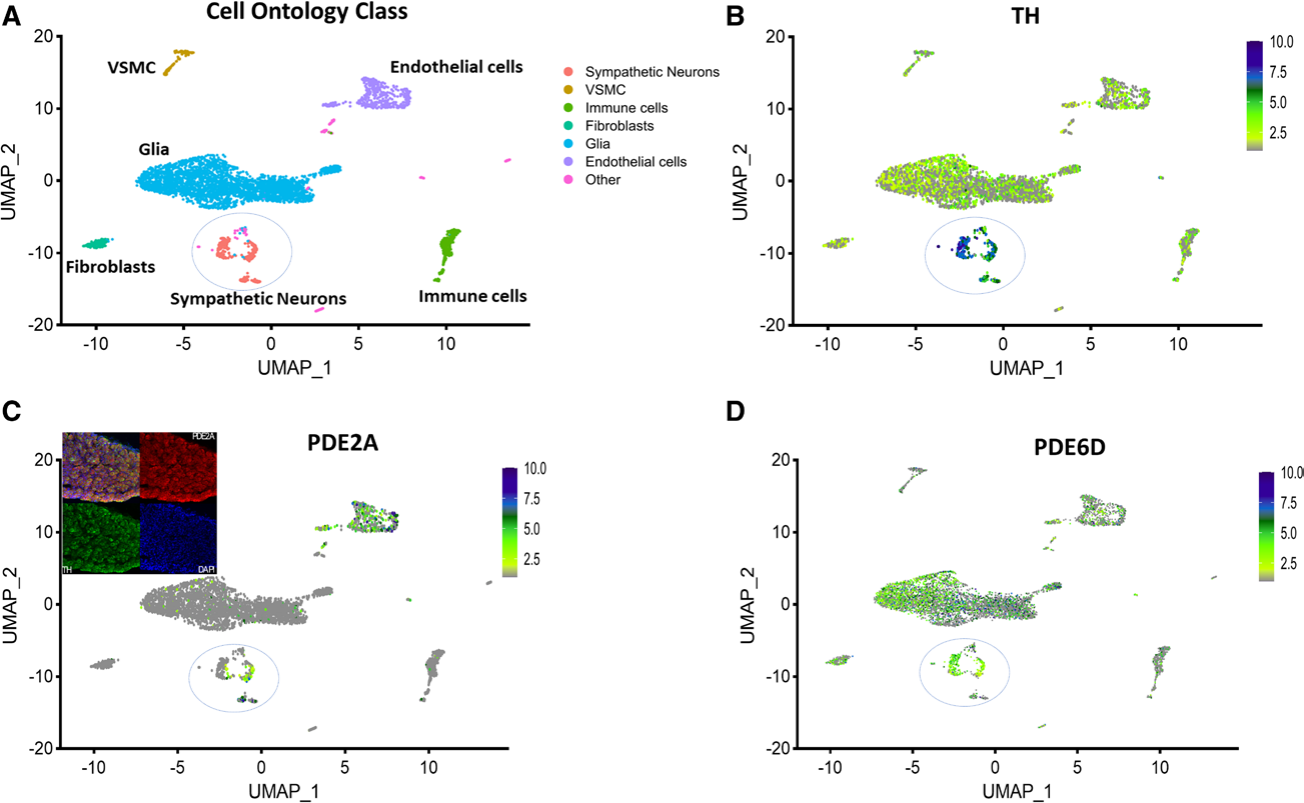
**PDE2A and PDE6 was expressed in sympathetic neurons (SNs). A**, Single-cell RNA sequencing on Wistar rat reveals multiple clusters of cell transcriptomes. **B**, SNs marker tyrosine hydroxylase (TH) was shown to be strongly expressed in SN clusters. **C**, PDE2A was high expressed in SN and endothelia cell (positive control) clusters. Inserted picture: immunohistochemistry of Wistar Left stellate ganglia cryosection confirms PDE2A (red) colocalize with SN marker TH (green). Nuclei were stained with DAPI (blue). **D**, PDE6D was high expressed in SN clusters.

Quantitative RT-PCR analysis of PDE2A in stellate ganglia tissue from 4-week-old SHR rats has shown significantly higher PDE2A mRNA levels compared with tissue from age-matched normal rats.^20^ Here, we show using single-cell RNA sequencing analysis that stellate ganglia from 4-week Wistar rats includes a heterogeneous cell population (Figure 1A). We also observed in both normal neurons and those from aged matched SHRs that several PDE gene families (eg, PDE2A, PDE6D, PDE7A, PDE9A) were highly expressed (Figure S1). We confirmed expression of PDE2A and PDE6D (Figure 1C and 1D) in sympathetic neuron (SN) clusters that co-localized with the sympathetic marker TH (tyrosine hydroxylase, Figure 1B), a finding that was further confirmed by immunofluorescence staining of cryosection generated from Wistar left stellate ganglia (PDE2A in red colocalized with the SN marker TH in green, Figure 1C insert).

### PDE2A Inhibition Reversed the Attenuated cAMP Signal at the OMM in Sympathetic Neurons From Young SHR

**Figure 2.**
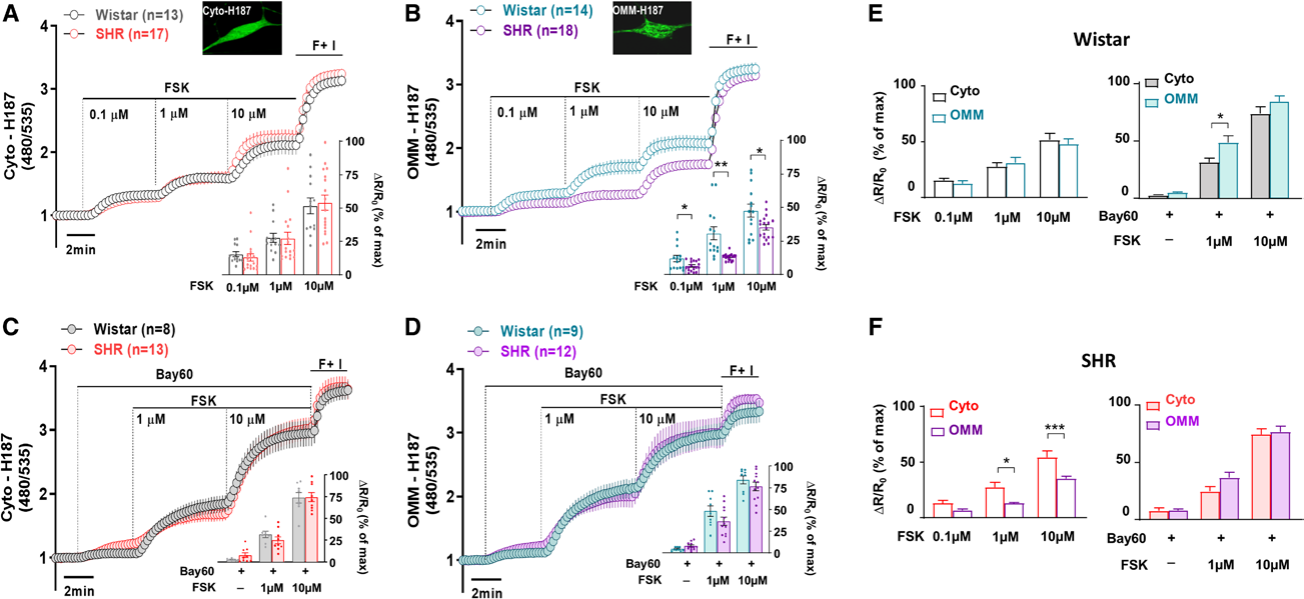
**Comparison of cAMP (cyclic adenosine 3’,5’-monophosphate) signals at the cytosol and outer mitochondrial membrane (OMM) levels in cardiac sympathetic neurons from 4-wk Wistar and spontaneously hypertensive rat (SHR).** Neurons transfected with cytosolic or OMM-targeted cAMP sensor, cyto-H187, or OMM-H187. Saturation of the sensor was achieved by 25 μM Forskolin (FSK) and 100 μM IBMX (F+I). **A** and **B**, Representative kinetics change of cytosolic (**A**) and OMM (**B**) cAMP levels in response to increasing concentrations of FSK (0.1, 1, 10 μmol/L) in SHRs and Wistar rats. (Inset) Average±SEM of percentage FRET changes (% of max). **C** and **D**, Representative kinetics change of cytosolic (**C**) and OMM (**D**) cAMP levels in response to PDE2A inhibitor Bay 60-7550 (1 μmol/L) alone and with increasing concentrations of Fsk (1, 10 μmol/L) in SHRs and Wistar rats. (Inset) Average±SEM of percentage FRET changes (% of max). **E**, Comparison of cytosolic and OMM cAMP levels in response to increasing concentrations of Fsk before (left) and after (right) PDE 2A inhibition with Bay 60-7550 (1 μmol/L) in Wistar rats. **F**, Comparison of cytosolic and OMM cAMP levels in response to different concentrations of Fsk before (left) and after (right) PDE 2A inhibition with Bay 60-7550 (1 μmol/L) in SHRs. *P<0.05, ***P<0.001, 1-way ANOVA. In each case, neurons were derived from 3 or more rats.

Mitochondria play a major role in the calcium transient difference observed between SN in SHRs and normal rats.^3^ Given that mitochondrial PDE2A2 regulates local cAMP levels,^28^ we directly explored whether compartmentalized cAMP signatures were different in SHR neurons. We used a FRET-based sensor, Epac-S^H187^ (H187) and its OMM-targeted variant OMM-H187, to monitor cAMP changes.^29^ Increasing doses of the broad-spectrum adenylate cyclase activator forskolin (Fsk, 0.1, 1, 10 µmol/L) produced matching increases in cAMP in both the cytosol and OMM (Figure 2A and 2B). Saturation of the sensor was achieved by using 25 μmol/L Fsk and the broad-spectrum PDE inhibitor 3-isobutyl-1-methylxanthine (IBMX, 100 μmol/L) at the end of each experiment. There was no difference in the cAMP levels measured in response to Fsk in the cytosol between SHR and Wistar neurons (Figure 2A). However, at the OMM, cAMP levels achieved by Fsk were significantly reduced in SHRs at all 3 concentrations (Figure 2B), suggesting selective local impairment of cAMP signaling at the OMM in the SHR neurons.

PDE2A has been shown to localize at the mitochondria.^28^ To test whether PDE2A is responsible for the differences in cAMP levels at the surface of mitochondria from SHR SNs, neurons were pretreated with the selective PDE2A inhibitor Bay 60-7550 (1 μmol/L). In the presence of Bay 60-7550, Fsk-induced cAMP responses were not different in the cytosol (Figure 2C), but the difference in the Fsk-induced cAMP increases at the OMM was abolished (Figure 2D).

We also compared cAMP responses in cytosolic and OMM domains in the same strain. In the Wistar SNs, the cAMP response to Fsk was not different at the cytosol and OMM level (Figure 2E, left), while the cAMP response was enhanced at OMM following PDE2A inhibition with 1 µM Fsk, but not in the presence of 10 µM Fsk (Figure 2E, right), suggesting PDE2A is functionally expressed at the OMM, and the cytosol. This result is consistent with the findings reported in HeLa cells.^29^ In contrast, in SHRs, the cAMP response to Fsk was reduced at the OMM compared with the cytosol (Figure 2F, left). These differences were rescued by inhibition of PDE2A (Figure 2F, right), suggesting cAMP signaling in the SHR was attenuated at the OMM as a consequence of increased local activity of PDE2A.

### PDE2A2 Regulation of cAMP at the OMM in Sympathetic Neurons

**Figure 3.**
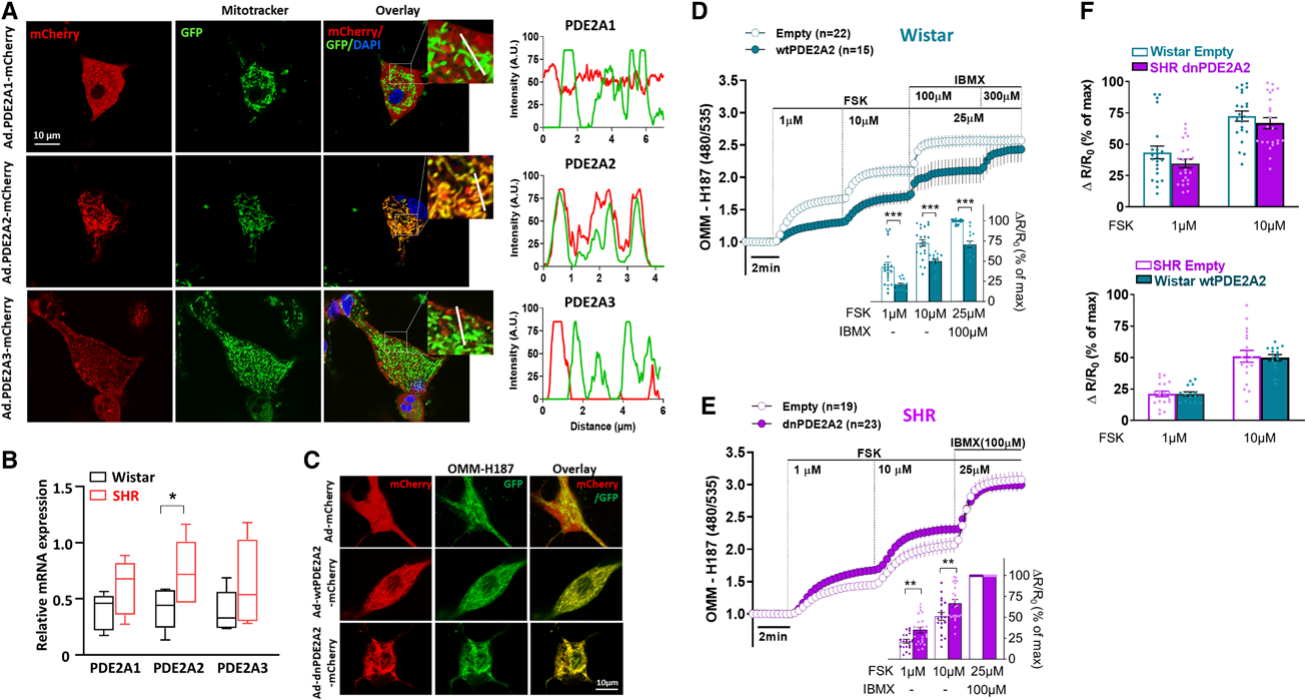
**PDE2A2 is located on the mitochondria and modulates outer mitochondrial membrane (OMM)-cAMP (cyclic adenosine 3’,5’-monophosphate) production in sympathetic neurons. A**, Localization of PDE2A1-mCherry, PDE2A2-mCherry, and PDE2A3-mCherry in cultured cardiac sympathetic neurons (SNs) derived from 4-wk Wistar rat stellate ganglion labeled with Mitotracker Green. Scale bar: 10 µm. Right: the fluorescence intensity profile for the Mitotracker Green (green line) with PDE2A1-mCherry (top), PDE2A2-mCherry (middle), and PDE2A3-mCherry (bottom) along with the line shown in the overlay images from **A**. **B**, Quantitative RT-PCR analysis of PDE2A1-3 mRNA levels in stellate ganglia tissue of 4-wk Wistar rats and SHRs. Data are shown as average±SEM. *P<0.05 by unpaired *t* test. **C**, Images showing single stellate neurons infected with Empty, wtPDE2A2 or dnPDE2A2 virus, which co-transduced with OMM-H187 sensor. Only transduced mCherry (red) neurons were selected to measure the forster resonance energy transfer (FRET) cyclic adenosine 3’,5’-monophosphate (cAMP) concentration. **D**, Gene transfer of adenovirus of wild-type PDE2A2 (wtPDE2A2) to the Wistar SNs reduced the Forskolin (FSK) enhanced OMM cAMP levels compared with empty victor Ad.mCherry. wtPDE2A2 transduced group also need extra IBMX (300 µmol/L) to saturation of the sensor. (Inset) average±SEM of percentage FRET changes (% of max). **E**, Overexpression of dominant-negative PDE2A2 virus (dnPDE2A2) in SNs from SHR enhanced the FSK affection at OMM cAMP levels compared with the empty victor Ad.mCherry. (Inset) Average±SEM of percentage FRET changes (% of max). **F**, Gene transfer of dnPDE2A2 in SHR neurons enhanced FSK-induced OMM-cAMP levels (top graph), which was no different from OMM-cAMP levels observed in healthy Wistar rats. Gene transfer of wtPDE2A2 in Wistar neurons mimic the SHR phenotype (bottom graph), produced same amount of OMM-cAMP levels induced by FSK. **P<0.01, ***P<0.001, 1-way ANOVA. In each case, neurons were derived from 3 or more rats. n indicates number of SNs.

To determine which PDE2A isoform regulates cAMP at the OMM domain, we first infected mCherry-labeled adenovirus expressing PDE2A1, PDE2A2, and PDE2A3 to the cultured Wistar cardiac SNs. Mitochondria were stained with MitoTracker Green (Figure 3A). PDE2A2-mCherry was distinctly located at the mitochondria, whereas PDE2A1-mCherry was present in the cytosolic, and PDE2A3-mCherry localized predominantly to the plasmalemma. These observations are consistent with the previous reports showing PDE2A isoforms have distinct subcellular locations in ventricular myocytes^28^ and in HEK cells.^13^ To clarify whether these isoforms are differentially expressed in SHR SNs as compared with normal SNs, quantitative RT-PCR analysis of PDE2A1-3 mRNA levels in stellate ganglia tissue of 4-week Wistar rats and SHRs was performed. Only mRNA expression for PDE2A2 in SHR neurons showed significant enhancement (n=6 in each group, *P<0.05; Figure 3B) when compared with neurons from normotensive Wistar rats.

To directly test the hypothesis that PDE2A2 modulates cAMP signaling in neurons at the OMM, we overexpressed PDE2A2 using a viral vector (Ad. wtPDE2A2-mCherry) in cultured Wistar neurons, or a dominant-negative PDE2A2 (a catalytically dead mutant of PDE2A2, Ad. dnPDE2A-mCherry.) in SHRs neurons. The empty vector (Ad.-mCherry) was used as a control in both cell types. Fluorescence microscopy detected mCherry expression in stellate neurons after transduction with the virus and OMM-targeted cAMP FRET reporter OMM-H187 (Figure 3C). Since not all neurons expressed mCherry, only transduced neurons were selected for FRET analysis of the cAMP levels. In the Wistar neurons, overexpression of wtPDE2A2 significantly diminished the Fsk-induced enhancement of cAMP detected with OMM-H187 compared with the neurons transduced with the empty vector (Figure 3D). Moreover, Wistar SNs, transduced with the wtPDE2A2 vector required a higher concentration of IBMX (300 µmol/L) to reach saturation compared with the empty vector control (100 µmol/L IBMX; Figure 3D). In contrast, expression of dnPDE2A2 in SHR neurons resulted in increased Fsk-induced cAMP at the OMM (Figure 3E), to a level similar the response detected at the OMM in native Wistar’s SNs (Figure 3F, top), SHR empty virus was similar to Wistar overexpression of wtPDE2A2 (Figure 3F, bottom). Taken together, these results indicate that overexpressing PDE2A2 in healthy neurons could mimic the disease phenotype and recapitulate the reduction of OMM-cAMP observed in SHR neurons, whereas expression of the catalytically inactive PDE2A2 mutant, by displacing the endogenous active PDE2A2, can restore in the diseased neurons the Fsk-induced cAMP response at the OMM to levels similar to those observed in healthy SNs.

### Enhancement of PKA Activity at the OMM of Sympathetic Neurons From Young SHR Is Reversed by PDE2A Inhibition

**Figure 4.**
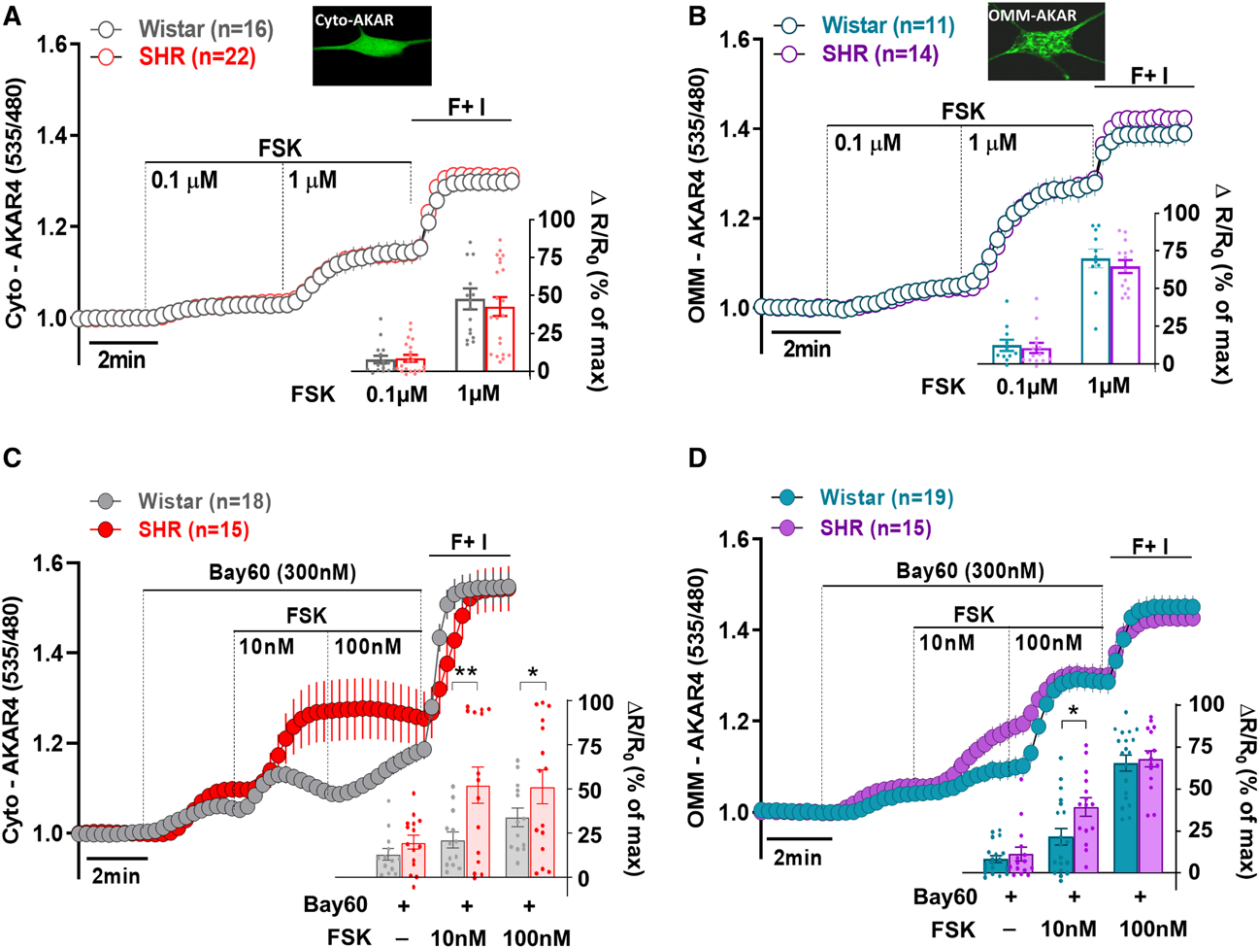
**Comparison of PKA (protein kinase A) signals at the cytosol and outer mitochondrial membrane (OMM) levels in cardiac sympathetic neurons from 4-wk Wistar and spontaneously hypertensive rats (SHRs).** Neurons transfected with cytosolic or OMM-targeted PKA sensor, cyto-AKAR4 or OMM-AKAR4. Saturation of the sensor was achieved by 25 μmol/L Forskolin (FSK) and 100 μmol/L IBMX (F+I). **A** and **B**, Representative kinetics change of cytosolic (**A**) and OMM (**B**) PKA levels in response to increasing concentrations of FSK (0.1, 1 μmol/L) in SHRs and Wistar rats. (Inset) Average±SEM of percentage FRET changes (% of max). **C** and **D**, Representative kinetics change of cytosolic (**C**) and OMM (**D**) PKA levels in response to Bay 60-7550 (300 nmol/L) alone and with increasing concentrations of FSK (10, 100 nmol/L) in SHRs and Wistar rats. (Inset) Average±SEM of percentage FRET changes (% of max). *P<0.05, **P<0.01, ***P<0.001, 1-way ANOVA. In each case, neurons were derived from 3 or more rats.

To determine whether the cAMP downstream effector PKA is also affected by the increase local activity of PDE2A at the mitochondria, we used soluble and OMM-targeted versions of the FRET-based sensor AKAR4 to measure PKA-dependent phosphorylation. Saturation of the sensor was achieved by using 25 μmol/L Fsk and 100 μmol/L IBMX at the end of each experiment. Increasing doses of Fsk (0.1, 1 µmol/L) resulted in enhanced PKA-dependent phosphorylation of AKAR4 in both the cytosol and OMM. In the cytosol, PKA-dependent phosphorylation was increased in response to Fsk at both concentrations (Figure 4A) with no difference in responses between cells from 2 strains. Since PKA activity is mostly dependent on cAMP, we asked the question whether the attenuated cAMP response at the OMM of SHR neurons would result in reduced PKA-dependent phosphorylation at this site. Surprisingly, no difference in AKA4 phosphorylation was detected at the OMM of Wistar’s or SHR neurons (Figure 4B), suggesting other factors might impact on the level of PKA-dependent phosphorylation.

Since PDE2A is responsible for the attenuated cAMP signal in sympathetic neurons from young SHR, we blocked PDE2A with Bay60-7550 to eliminate the effect of the cAMP differences to PKA. Bay60 itself increased the baseline PKA-dependent phosphorylation levels both in the cytosol and at the OMM, although there was no difference between the SHR and Wistar (Figure 4C and 4D). Given that 1 µmol/L Fsk under PDE2A inhibition saturated the PKA sensor, we reduced the Forskolin concentration to 10 and 100 nmol/L. After PDE2A inhibition, higher PKA-dependent phosphorylation was recorded in the cytosol at both Fsk concentrations. At the OMM, the larger PKA activity was detected only at the lower Fsk concentration (Figure 4C and 4D).

### Are Protein Phosphatases Responsible for the Enhanced PKA Action in Sympathetic Neurons From Young SHR?

**Figure 5.**
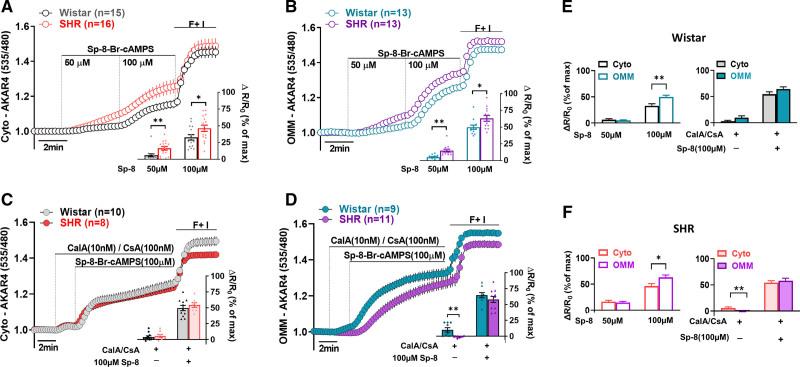
**Protein phosphatases are responsible for the different AKAR4 responses between spontaneously hypertensive rat (SHR) and Wistar sympathetic neurons in the cytosol and outer mitochondrial membrane (OMM). A** and **B**, AKAR4 signals in response to PDE-resistant cAMP analogue Sp-8-Br-cAMPS (50, 100 μmol/L) were significantly enhanced in the SHRs on both cytosolic (**A**) and OMM (**B**) level. (Inset) Average±SEM of percentage forster resonance energy transfer (FRET) changes (% of max). Inhibition of protein phosphatases with CalA (50 nmol/L)/CsA (200 nmol/L) eliminated the different AKAR4 response to Sp-8-Br-cAMPS between SHR and Wistar in the cytosol (**C**) and the OMM (**D**). (Inset) Average±SEM of percentage FRET changes (% of max). **E**, Comparison of cytosolic and OMM PKA levels in response to Sp-8-Br-cAMPS before (left) and after (right) phosphatases inhibition with CalA (50 nmol/L)/CsA (200 nmol/L) in Wistar rats. **F**, Comparison of cytosolic and OMM PKA levels in response to Sp-8-Br-cAMPS before (left) and after (right) phosphatases inhibition with CalA (50 nmol/L)/CsA (200 nmol/L) in SHRs. *P<0.05, **P<0.01, ***P<0.001, 1-way ANOVA. In each case, neurons were derived from 3 or more rats.

To measure PKA-dependent phosphorylation independently of PDE activity, we treated the neurons with the PDE-resistant cAMP analogue Sp-8-Br-cAMPS (50, 100 µmol/L). Sp-8-Br-cAMPS significantly enhanced the AKAR4 signal in the cytosol (Figure 5A) and at the OMM (Figure 5B) in SHR neurons at both concentrations of analogue used, indicating PKA-dependent phosphorylation increases in the SHR. Maintaining the proper balance of phosphorylation not only depends on the appropriate addition but also the appropriate removal of phosphates.^30^ Therefore, we investigated whether protein phosphatases were responsible for the different level of PKA-dependent phosphorylation of the PKA reporters, we inhibited PP2A (protein phosphatases 2A) and PP1 (protein phosphatases 1) with calyculin A, and PP2B (protein phosphatases 2B) with cyclosporine A, before the application of Sp-8-Br-cAMPS (100 µmol/L). As shown in Figure 5C and 5D, calyculin A/cyclosporine A alone slightly raised the phosphorylation level of AKAR4 at both cytosol and OMM, and this change was not observed in the SHR neurons at the OMM. Furthermore, phosphatase inhibition abolished the differences of Sp-8-Br-cAMPS stimulation, which was observed in both compartments between SHR and Wistar. We also noted that at the OMM AKAR4 levels were higher compared with the cytosol with increasing concentration of Sp-8-Br-cAMPS (100 µmol/L) for both Wistar (Figure 5E, left) and SHRs (Figure 5F, left). These differences were abolished by inhibition of phosphatases in Wistar (Figure 5E, right) and SHR neurons (Figure 5F, right).

### Downregulation of cGMP Signaling at the OMM in Sympathetic Neurons From Young SHR

**Figure 6.**
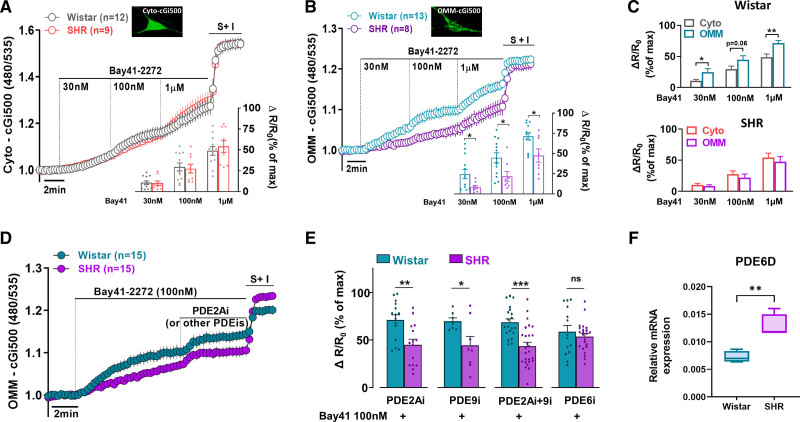
**Comparison of sGC/cGMP signals at the cytosol and OMM levels in cardiac sympathetic neurons from 4-week Wistar and SHR.** Neurons transfected with cytosolic or OMM-targeted cGMP sensor, cyto-cGi500 or OMM-cGi500. Saturation of the sensor was achieved by 20 μmol/L SIN-1 and 100 μmol/L IBMX (S+I). **A&B**: Representative kinetics change of cytosolic (**A**) and OMM (**B**) cGMP levels in response to increasing concentrations of Bay41-2272 (30, 100, 1000 nmol/L) between SHRs and Wistar rats. (**Inset**) Average ± SEM of percentage FRET changes (% of max). **C**: Comparison of cytosolic and OMM cGMP levels in response to increasing concentrations of Bay41-2272 within Wistar (**top**) and SHR (**bottom**) SNs. **D**: Effect of PDE2A or the other cGMP specific PDEs (PDE9, PDE6) on the Bay41 induced different of cGMP production in SHRs and Wistar rats. **E**: PDE6 inhibition could abolished the differences of the of cGMP induced by Bay41 at OMM between SHRs and Wistar rats, whereas inhibition of PDE2A, PDE9, or combination of PDE2A & PDE9 failed to rescue the differences. **F**: Quantitative RT-PCR analysis of PDE6D mRNA levels in stellate ganglia tissue of 4-week Wistar rats and SHRs. Data are shown as Average ± SEM. *P < 0.05, **P < 0.01, ***P < 0.001, ns: not significant. One-way ANOVA or *t* test to compare the means of 2 groups. In each case, neurons were derived from 3 or more rats.

To clarify whether cGMP signaling is impaired in SHR neurons, the cGMP FRET-based sensor cGi500^25^ was targeted at the cytosol and OMM. The nitric oxide–independent sGC (soluble guanylate cyclase) stimulator Bay 41-2272 was used to induce cGMP synthesis. Saturation of the sensor was achieved by using nitric oxide donor 3-morpholinosydnonimine (SIN-1, 20 μmol/L) and IBMX (100 μmol/L) at the end of each experiment. Increasing concentrations of BAY41-2272 (Bay41 30, 100, 1000 nmol/L) produced matching increases in cGMP in both the cytosol (Figure 6A) and OMM (Figure 6B). There was no difference between strains in the cytosol response to Bay41 (Figure 6A), whereas cGMP was significantly reduced at the OMM in SHR compared with Wistar neurons at all 3 concentrations (Figure 6B). cGMP responses detected by cytosolic and OMM sensors were also compared within the same strain. In the Wistar neurons, Bay41 increased cGMP production higher at the OMM than cytosol (Figure 6C, top), while no difference was observed between the 2 compartments in the SHR’s (Figure 6C, bottom).

According to the results obtained by single-cell gene sequencing (Figure S1), we inhibited the major cGMP-specific PDEs present in SNs: PDE9A, PDE6D, and PDE2A, to investigate, which PDE is responsible for the decreased cGMP response at OMM of SHR. Neurons were treated with PDE inhibitors after application of Bay41 (100 nmol/L, Figure 6D). In the presence of Bay41, inhibition of PDE2A with Bay60-7550 (1 μmol/l), or PDE9 with PF-04447943 (PF, 100 nmol/L), and the combination of PDE2A and PDE9 inhibition all failed to abolish the differences in the cGMP response at OMM between SHRs and Wistar rats (Figure 6E). However, inhibition of PDE6 with Zaprinast at 0.5 μmol/L normalized the cGMP response at OMM in the SHR neurons (Figure 6E, far right). To date, no selective inhibitors of PDE6 have been reported. Zaprinast is a phosphodiesterase inhibitor, selective for PDE6, 5, 11, and 9 (IC values are 0.15, 0.76, 12.0, and 29.0 μmol/L, respectively). We used a concentration of 500 nmol/L, which is lower than the IC_50_ for other PDEs, supporting the idea that most of the cGMP response between the different strains of neurons at the OMM is due to the inhibition of PDE6.^26^ Quantitative RT-PCR analysis of PDE6D confirmed mRNA levels in stellate ganglia tissue of 4-week Wistar rats and SHRs, where expression for PDE6D was enhanced in SHR neurons (n=3 in each group, **P<0.01; Figure 6F).

## Discussion

The present study investigated the dynamic changes of cAMP-PKA and cGMP signaling in microdomains of postganglionic SNs from the prehypertensive SHR. Here, we report several novel findings. First, cAMP levels at the OMM microdomain is decreased in diseased stellate neurons. PDE2A inhibition reverses this response. Second, the PDE2A isoform PDE2A2, which is located at the OMM, can regulate local cAMP levels in sympathetic neurons. Third, there is greater PKA-dependent phosphorylation in the cytosol and at the OMM in SHR neurons. This response was inhibited by protein phosphatases. Fourth, in diseased neurons activation of the cGMP pathway is impaired at the level of the OMM. Inhibition of PDE6 restored cGMP responsiveness.

There is a growing body of literature demonstrating that PDEs contribute to the compartmentalization of cyclic nucleotide signaling.^31–33^ Compelling data support the formation of molecular complexes (signalosomes) in distinct cellular compartments that influence cyclic nucleotide signaling in cardiomyocytes^19^ and neurons.^34,35^ Why is this important in hypertension? Emerging evidence has reported that a significant component of the sympathetic hyper-responsiveness in the prehypertensive SHR is linked to impaired regulation of cyclic nucleotides.^14,27,36^ Specifically, high levels of PDE2A have been reported in both stellate neurons from the SHR and in patients following stellectomy due to sympathetic dysautonomia.^20^ Overexpression of PDE2A with an adenoviral vector into healthy stellate neurons recapitulates the sympathetic phenotype, which is associated with low levels of cGMP, resulting in enhanced [Ca^2+^]_i_ and increased neurotransmission. These responses were blocked by PDE2A inhibition or exogenous expression of the dominant negative of PDE2A,^20^ suggesting this might be a therapeutic target. Moreover, in pulmonary hypertension, PDE2A inhibition also elicits pulmonary dilation, prevents pulmonary vascular remodeling, and reduces right ventricular hypertrophy.^37^ However, the precise site where PDE2A acts in sympathetic neurons has not been established.

This study demonstrates that following addition of forskolin, PDE2A inhibition can equalize the differences in cAMP generation at the OMM between SHR and Wistar neurons (Figure 2D). One interesting observation is that the addition of the PDE2A inhibitor Bay-60 increases Fsk-induced cAMP to a greater extent in the SHR at the OMM, although this is not the case for global cytosolic cAMP (Figure 2F). Since PDE2A2 is overexpressed in SHR neurons, this may explain the differences in cAMP levels observed between cytosol and mitochondria of SHR neurons (see Figure 3A and 3B).

PDE2A expresses as 3 splice variants PDE2A1, PDE2A2, and PDE2A3.^38^ These isozymes differ at their N-terminal residues, allowing different subcellular locations: PDE2A1 is cytosolic, whereas PDE2A2 and PDE2A3 are membrane-associated.^39^ Although we did not quantify the protein localization of these isoforms due to the paucity of tissue and poor specificity of antibodies, we nevertheless could visualize mitochondrial co-localization under fluorescence microscopy (Figure 3A). We detected that PDE2A2 is distinctly located at the mitochondria, PDE2A1 is in the cytosol, and PDE2A3 localizes predominantly to the plasmalemma. PDE2A2 mRNA expression showed significant enhancement in SHR SNs. Interestingly, using transmission electron microscopy and immunogold labeling, Monterisi et al^28^ further confirmed that PDE2A2 localizes to the OMM and inner mitochondrial membrane, whereas this enzyme appears to be largely excluded from the intracristae space and from the matrix in primary neonatal rat ventricular myocytes. They also observed that PDE2A2 regulates local cAMP levels and PKA-dependent phosphorylation of Drp1. Whether this pathway is present in sympathetic neurons remains to be tested. Since cAMP can pass through the OMM, it is therefore possible that PDE2A2 could regulate cAMP levels detected at the OMM.^40^

To test this hypothesis, we found that overexpressing PDE2A2 in healthy stellate neurons reduces the level of cAMP detected by an OMM-localized FRET sensor (Figure 3D), recapitulating the phenotypes observed in stellate neurons from SHR rats. Whereas SHR neurons expressing a dominant negative form of the PDE2A2 isoform showed increases in cAMP detected at the OMM following addition of forskolin (Figure 3E). This suggests that in these neurons, cAMP at the OMM is directly under the specific control of PDE2A2.

From this study, we observed that the cAMP signaling through its downstream effector PKA at the OMM are normal following forskolin stimulation (Figure 4B) in the SHR SNs, despite the impairment of cAMP. However, the greater PKA phosphorylation at both the cytosol and OMM following PDE2A inhibition (Figure 4C and 4D), or direct activation of PKA (Figure 5A and 5B), could conceivably compensate for the impaired cAMP dynamics in this spatial domain. This might be due to diminished phosphatase activity in SHR neurons in the cytosol and around the OMM. We also detected that PKA activity is predominately enhanced at OMM domain in both strains after stimulating PKA with Sp-8-Br-cAMP (Figure 5E and 5F, left). The addition of a phosphatase inhibitor had little effect on PKA activity in the SHR due to diminished phosphatase activity, however it further enhanced PKA activity in the Wistar (Figure 5C and 5D). Differences of PKA activity between the cytosol and the OMM were abolished in both strains (Figure 5E and 5F, right). This is consistent with other findings reporting that phosphatases-dependent dephosphorylation of PKA targets is attenuated at the OMM in neonatal cardiomyocytes^29^ and cancer cells.^41,42^ Of interest, protein phosphatases have been implicated in a wide variety of illnesses,^43^ including hypertension.^44^ Both PP1 and PP2A are part of the serine/threonine phosphoprotein phosphatase (PPP) family and account for the vast majority of eukaryotic phosphatase activity via cAMP-dependent inhibition of PP1 (phosphorylates DARPP-32 at Thr-34) and PP2 (phosphorylates the B56δ subunit).^30^ Whereas, PP2B counteracts the actions of PKA on mitochondrial morphology.^45^ The detailed mechanisms of how these components affect the sympathetic neurons in the SHR remain to be further discovered.

There are 2 distinct pathways that can activate cGMP: the NP (natriuretic peptide)/pGC (particulate guanylyl cyclase)/cGMP pool is formed at the plasma membrane, and nitric oxide/sGC/cGMP pool formed in the cytosol and/or caveolin-rich membrane domains.^46^ In cardiomyocytes, pGC/cGMP is tightly controlled by PDE2A and PDE9, while the sGC/cGMP pool is predominantly regulated by PDE5 and PDE3.^19^ In vascular smooth muscle cells, the pGC/cGMP pool is controlled by PDE5 and PDE3, and the sGC/cGMP pool is mainly regulated by PDE3.^47^ We have recently demonstrated that PDE2A plays a key role in modulating the efficacy of BNP via NP/pGC/cGMP signaling on the calcium current/ transient and neurotransmission in SNs,^20^ presumably via PDE2A3, which localizes predominantly to the plasmalemma. In this study, when we directedly activated sGC in neurons, we observed that cGMP production was attenuated at the OMM in the SHRs when compared with normal rats. This difference was rescued by inhibition of PDE6, but not by PDE9 and PDE2A, which were also highly expressed in the neuron. To the best of our knowledge, this is the first report showing in the sympathetic neurons, a sGC/cGMP pool is predominantly regulated by PDE6.

### Perspectives

In conclusion, this work suggests that modulation of abnormal cyclic nucleotide signaling at the OMM can be achieved by inhibition of local phosphodiesterase activity in sympathetic neurons during the early stages of hypertension. Whether the OMM is a therapeutic target to ameliorate sympathetic impairment remains to be established.

## Article Information

### Author Contributions

D. Li, K. Liu, C. Robertson, O.C. Robertson, A. Tarafdar, and N. Li performed and analyzed experiments. H. Davis analyzed the single-cell RNA sequencing data. D. Li, K. Liu, and H. Davis produced figures. K. Lefkimmiatis provided the FRET sensor. M. Zaccolo provided PDE2A virus and contributed experimental design. D. Li and D.J. Paterson designed the project and co-wrote the article.

### Sources of Funding

We acknowledge the British Heart Foundation (RG/17/14/33085), the British Heart Foundation Center of Research Excellence for funding this work. HD supported by the Wellcome Trust OXION Program (102161/Z/13/Z). N. Li was supported by China Scholarship Council and the Chinese Academy of Medical Sciences & Peking Union Medical College (CAMS/PUMC) Innovation Fund for Medical Science (CIFMS).

### Disclosures

None.

## Supplementary Material


