# Supplementary material for: Abnormal Cyclic Nucleotide Signaling at the Outer Mitochondrial Membrane In Sympathetic Neurons During the Early Stages of Hypertension
Source: Hypertension. 2022 May 4;79(7):1374–84. doi: 10.1161/HYPERTENSIONAHA.121.18882 (PMC9172895; doi:10.1161/HYPERTENSIONAHA.121.18882)
Supplement: Supplementary file 1 [file hyp-79-1374-s001.pdf]

Online Supplement:

Abnormal cyclic nucleotide signaling at the outer mitochondrial membrane in sympathetic neurons during the early stages of hypertension

Dan Li[1*†], Kun Liu[1*], Harvey Davis[1,2], Calum Robertson[1], Oliver C. Neely[1], Adib Tarafdar[1], Ni Li[1,3], Konstantinos Lefkimmiatis[4,5], Manuela Zaccolo[1], David J. Paterson[1†]

1. Burdon Sanderson Cardiac Science Centre and BHF Centre of Research Excellence, Department of Physiology, Anatomy and Genetics, University of Oxford, Oxford, OX1 3PT, UK
2. Department of Neuroscience, Physiology and Pharmacology, University College London, London WC1E 6BT, UK
3. Chinese Academy of Medical Sciences Oxford Institute (COI), Nuffield Department of Medicine Research Building, University of Oxford, Old Road Campus, Oxford OX3 7BN, UK
4. Department of Molecular Medicine, University of Pavia, Pavia, Italy
5. Veneto Institute of Molecular Medicine, Padova, Italy

* These authors contributed equally to this work

† ***Correspondences:***
Dan Li & David J. Paterson
Burdon Sanderson Cardiac Science Centre
Department of Physiology, Anatomy and Genetics,
Sherrington Building, Parks Road,
Oxford, OX1 3PT, UK
Tel: +44 1865 272547,
E-mail: dan.li@dpag.ox.ac.uk & david.paterson@dpag.ox.ac.uk

Supplement Methods

Solutions and Materials: All chemicals were purchased from Sigma (St. Louis, MO) except Bay60-7550, purchased from Cayman Chemicals (Ann Arbor, MI), Sp-8-Br-cAMPS, from BioLog (Bremen, Germany), and MitoTracker Green, from Invitrogen (Invitrogen, MA). Experiments were performed using normal Tyrode's solution containing (in mM) 125 NaCl, 4.5 KCl, 20 HEPES, 11 Glucose, 1 MgCl2, 2 CaCl2. For cell culture, L-15 blocking medium was composed as follows: 96.8% L-15 medium, with 0.6% D-(+)-Glucose solution, 2mM L-glutamine, 100 units/ml penicillin, 100μg/ml streptomycin, and 10% fetal bovine serum. Plating medium was composed as follows: 90% L-15 medium, with 24mM NaHCO3, 38mM glucose, 50 units/ml penicillin, 50μg/ml streptomycin, 50ng/ml nerve growth factor, and 10% fetal bovine serum.

Primary Cultures of Dissociated Sympathetic Neurons. Sympathetic neurons were isolated and cultured using a previously published method and the media used for isolation were based on modification of those previously described[1]. Briefly, stellate ganglia (which provide the majority of cardiac sympathetic innervation) were excised under sterile conditions, and placed into cold Hank's Balanced Salt Solution. Any contaminating connective tissue was removed. Each ganglion was then cut into 6-8 pieces, and digested in 1mg/ml type IV collagenase (for 20 min), followed by 2mg/ml type I trypsin (25 min), both at 37°C. Stellate ganglia were washed twice in L-15 blocking medium, and twice in plating medium (each time for 5 min). Subsequently, a single-cell suspension was produced by manual trituration in 1ml pre-equilibrated plating medium. This suspension was plated onto poly-D-lysine/laminin coated 6mm cover slips, in 1.9cm^2 wells of plating medium (4 cover slips/well).

Single-cell RNA sequencing: A pooled single cell suspension of stellate ganglia cells from six animals per strain was prepared via enzymatic dissociation as described under cell culture methods. Following blockade of enzymatic activity via three washes in blocking solution, the cell solution was transferred to phosphate buffered saline. The cell solutions were immediately transferred to ice and transported to the Wellcome Trust Centre for Human Genetics (WTCHG) for scRNAseq via 10x genomics chromium (10x genomics, US) (Single Cell 3' v3) and Illumina hiseq 4000 (Illumina, US). This approach achieved 66-72K mean reads per cell and a sequencing depth of 53-55% per cell before filtering.

RNA extraction and qRT-PCR: Total RNA from sympathetic ganglia tissue from Wistar and SHRs was extracted using a RNeasy Protect Mini Kit (Qiagen) according to the manufacturer's instructions. For reverse transcription, first-strand cDNA was synthesized from 1 μg of total RNA with the iScript cDNA Synthesis Kit (Bio-Rad) according to the manufacturer's protocol. qRT-PCR was conducted in a total of 20 μl containing 10 μl of Taqman Universal PCR Master mix (Applied Biosystems), 2 μl of cDNA (10 ng/μl), 1 μl of 20× specific primers for Taqman Gene Expression Assays (NM_001143847.2 for PDE2A1, NM_031079.2 for PDE2A2, NM_001270604.2 for PDE2A3, Rn01400276_m1 for PDE6D, Rn00560865_m1 for B2M, Thermo Fisher Scientific), and 7 μl of DNase-free water. qRT-PCR was performed in a 96-well clear optical reaction plate 7000 apparatus (Applied Biosystems), and the thermal cycling conditions were 2 minutes at 50°C, 10 minutes at 95°C, followed by 40 cycles of 15 seconds at 95°C and 1 minutes at 60°C. All samples and standards were run as triplicates. Results were analyzed with the ABI Prism 7000 Sequence Detection System software (Applied Biosystems). Gene expression was normalized to B2M that was used as an internal control.

Adenovirus vector transduction: An adenoviral vector tagged carrying mCherry-tagged PDE2A1, PDE2A2, PDE2A3 (to study the localisation of PDE2A isoform in cells loaded with MitoTracker dye) or a catalytically inactive PDE2A2 (dnPDE2A2, for FRET and calcium transient measurement) was transduced into cultured cardiac sympathetic neurons. An adenoviral vector expressing only mCherry (Ad.CMV-mCherry) was used as a control. 5×10^7 pfu of adenoviral vector was used to infect neurons in a 4 well plate (1.9 cm^2/well, Nunc, Denmark). The virus containing medium was left in the well a maximum of 18 hours before changing to fresh medium. Experiments were performed after 2-3 days following gene transfer.

FRET imaging: Sympathetic neurons were transduced with adenoviral vectors encoding for the following different FRET-based sensors: cyto-EPACS^{H187}(H187, cytosolic cAMP sensor), OMM-H187[2] (cAMP sensor targeted to the OMM), cyto-AKAR4 (cytosolic sensor for PKA activity), OMM-AKAR4[3] (PKA-activity sensor targeted to the OMM), cyto- cGi500 (cytosolic sensor for cGMP) and OMM-cGi500[2,4] (cGMP sensor targeted to the OMM). Generation and amplification of the viral particles was outsourced to Vector Biolabs. The sensors containing medium was left in the well a maximum of 18 hours before changing to fresh medium. FRET imaging experiments were performed 2-3 days after transduction.

A neuron-containing coverslip was placed into a homemade open perfusion chamber (volume 100 µL). Cells were perfused with a gravity-fed perfusion system (MVC-801, MappingLab, UK) with a velocity of 1.5 mL/min. Imaging using a 40x oil-immersion objective was carried out at room temperature on an inverted Nikon microscope equipped with FRET imaging apparatus. This consisted of an OptoLED light source (Cairn Research Ltd.), a CoolSNAP HQ2 digital CCD camera (Photometrics), and DV2 beam-splitter (Photometrics) with 05-EM filter set; this set contains the specific set of emission filters for donor and acceptor fluorophore acquisition (dichroic mirror 505DCXR, donor emission of 480nm, and acceptor emission of 535nm; Chroma Technology Corp). Upon excitation at 430nm (every 15 seconds), changes in the 480 and 535 nm emission intensities were recorded (with background subtraction), and the 535/480nm ratio was calculated. The mean FRET response was plotted against time and expressed as the percentage of $\Delta R/R0$, where $\Delta R = R - R0$ and R0 is the mean of the four FRET ratios preceding the addition of the first drug, and R is the ratio at time = t seconds.

Immunofluorescence microscopy: Freshly isolated stellate ganglia were immediately transferred to 4% paraformaldehyde for 1-2 hours, after which the tissue was incubated overnight in 20% sucrose-PBS at 4ºC, before embedding in OCT compound (TissueTek). Tissue was then frozen and stored at -80ºC until cryosectioning the tissue as 12 µm sections. Slides were then permeabilized in 0.3% triton-X for 30 minutes at room temperature, before blocking for 2 hours in 1% BSA, 5% donkey serum. Sections were then incubated for 24 hours with primary antibodies, a mouse anti-TH (66334-1-Ig, ProteinTech, US) and a rabbit antibody against PDE2A (pAb, Thermo Fisher Scientific, PA1-31128) at 4ºC, followed by five 5 minutes washes in PBS and 2 hours incubation with the relevant secondary antibodies. Sections were subsequently washed 3 times in PBS, and incubated with DAPI/PBS for 5 minutes, before a final 2 washes in PBS. Slides were then mounted with 50% glycerol in PBS before imaging. Sections were imaged on a Zeiss LSM 880 Airy Scan Upright laser-scanning confocal microscope with a Plan-Apochromat 20x/0.8 M27 objective.

For mitochondria markers co-localization imaging, cultured stellate neurons were transduced with mCherry tagged Ad.PDE2A1, PDE2A2, PDE2A3 respectively for 3 days, then

incubated with 100 nmol/L MitoTracker Green (Invitrogen, M7514) for 5 min at 37°C. Images were acquired with an Inverted Nikon Eclipse Ti A1 Confocal Laser Microscope and processed with the image processing program ImageJ.

References

1. Li D, Lee C, Buckler K, Parekh A, Herring N, Paterson DJ. Abnormal Intracellular Calcium Homeostasis in Sympathetic Neurons From Young Prehypertensive Rats. *Hypertension*. 2012;59(3):642–649.

2. Brescia M, Chao Y-C, Koschinski A, Tomek J, Zaccolo M. Multi-Compartment, Early Disruption of cGMP and cAMP Signalling in Cardiac Myocytes from the mdx Model of Duchenne Muscular Dystrophy. *International Journal of Molecular Sciences*. 2020;21(19):7056.

3. Burdyga A, Surdo NC, Monterisi S, Di Benedetto G, Grisan F, Penna E, Pellegrini L, Zaccolo M, Bortolozzi M, Swietach P, Pozzan T, Lefkimmiatis K. Phosphatases control PKA-dependent functional microdomains at the outer mitochondrial membrane. *Proceedings of the National Academy of Sciences*. 2018;115(28):E6497–E6506.

4. Liu K, Li D, Hao G, McCaffary D, Neely O, Woodward L, Ioannides D, Lu C-J, Brescia M, Zaccolo M, Tandri H, Ajijola OA, Ardell JL, Shivkumar K, Paterson DJ. Phosphodiesterase 2A as a therapeutic target to restore cardiac neurotransmission during sympathetic hyperactivity. *JCI Insight*. 2018;3(9):e98694.

Figure S1

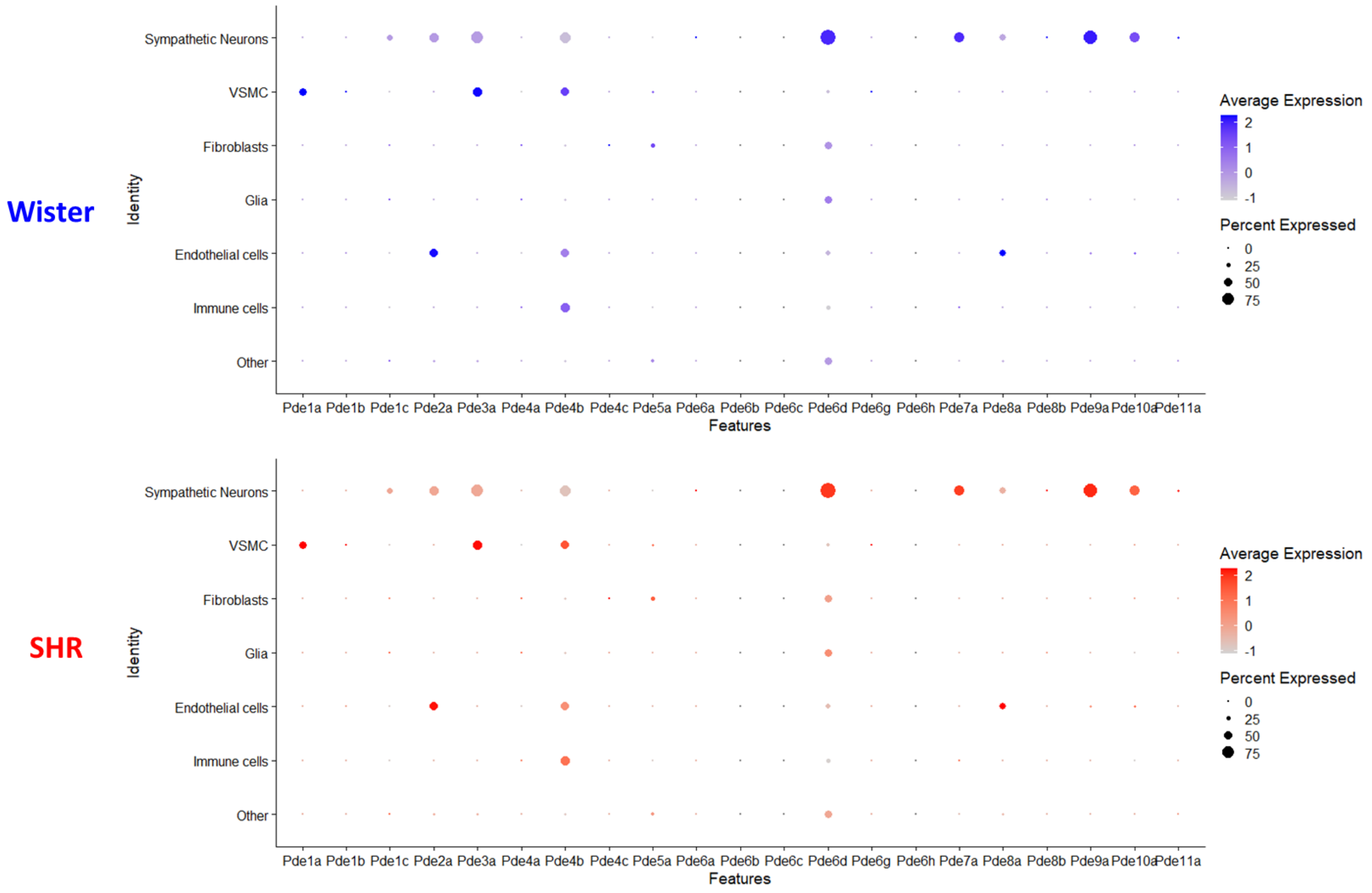

Figure S1: PDE gene families are shown to be expressed in SNs from Wistar (top) and SHR (bottom) single-cell RNA sequencing.

Figure S2

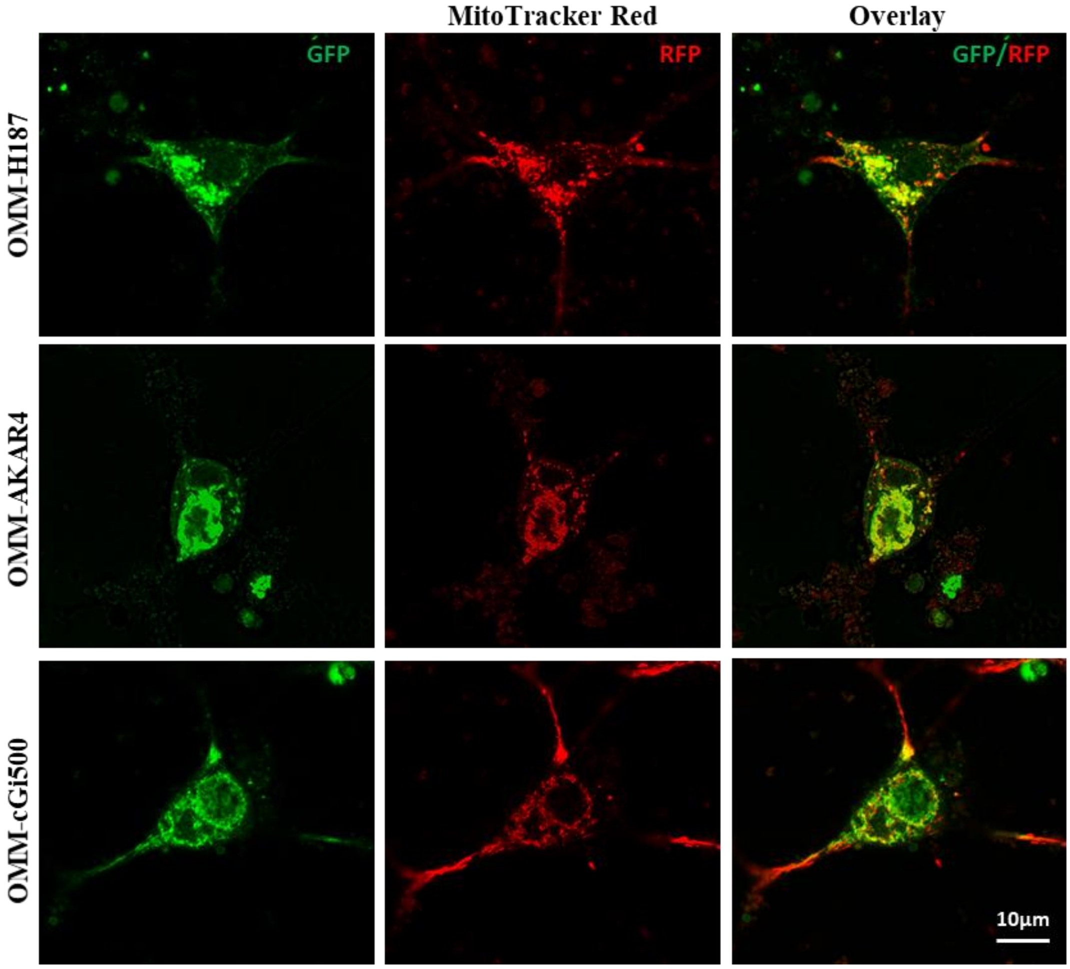

Figure S2: Colocalization of OMM FRET probes OMM-H187, OMM-AKAR4 and OMM-cGi500 with MitoTracker red in cultured cardiac sympathetic neurons derived from 4-week Wistar rat stellate ganglion. Scale bar: 10 µm.
